# Loss of buoyancy control in the copepod *Calanus finmarchicus*

**DOI:** 10.1093/plankt/fbz036

**Published:** 2019-10-14

**Authors:** Jonathan H Cohen, Kim S Last, Jack Waldie, David W Pond

**Affiliations:** 1 School of Marine Science and Policy, University of Delaware, 700 Pilottown Road, Lewes, DE, 19958, USA; 2 Scottish Association for Marine Sciences, Scottish Marine Institute, Oban, Argyll PA37 1QA, UK; 3 Institute of Aquaculture, University of Stirling, Stirlingshire, FK9 4LA, UK

**Keywords:** salinity, temperature, pressure, lipid phase transition, depth regulation

## Abstract

A mechanism is demonstrated that could explain large-scale aggregations of lipid-rich copepods in the surface waters of marine environments. Laboratory experiments establish that changes in salinity and temperature induce lipid-mediated buoyancy instability that entrains copepods in surface waters. Reduced hydrostatic pressure associated with forced ascent of copepods at fjordic sills, shelf breaks and seamounts would also reduce the density of the lipid reserves, forcing copepods and particularly those in diapause to the surface. We propose that salinity, temperature and hydrodynamics of the physical environment, in conjunction with the biophysical properties of lipids, explain periodic high abundances of lipid-rich copepods in surface waters.

## POTENTIAL MECHANISM DRIVING DENSE AGGREGATIONS OF CALANOID COPEPODS IN SURFACE WATERS

**Fig 1:**
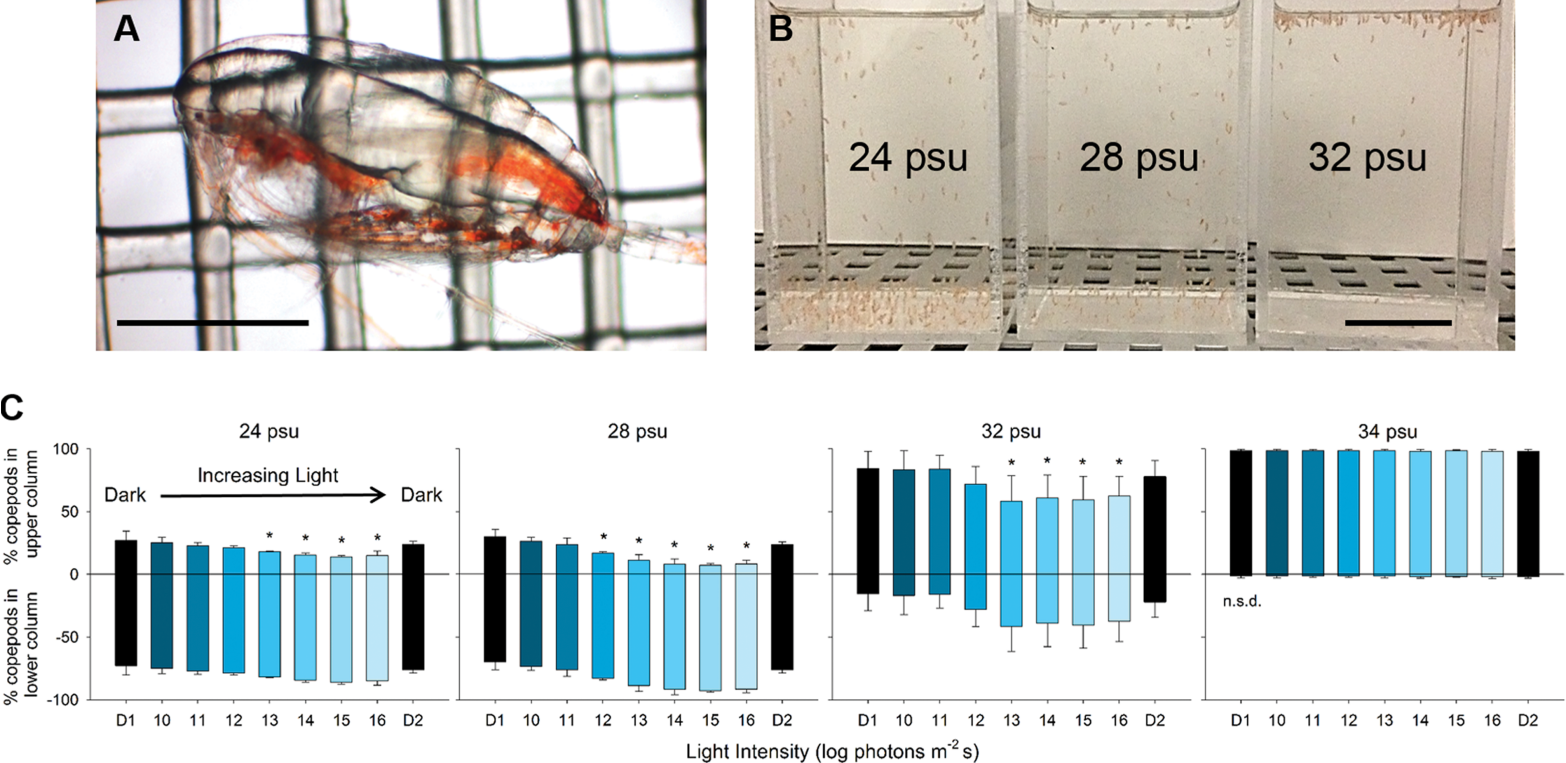
Salinity effects on CV-stage *Calanus finmarchicus* buoyancy at atmospheric pressure. (**A**) Lateral view of CV-stage *C. finmarchicus*, collected from Loch Etive, Scotland, UK, with a prominent oil sac. Scale bar = 1 mm. (**B**) Demonstration that acute salinity disrupts CV-stage *C. finmarchicus* buoyancy control. Following collection from Loch Etive (salinity 27–28 psu), copepods were placed in columns filled with GF/F-filtered 50-m-depth Loch Etive water adjusted to 24, 28 and 32 psu in darkness within an incubator (11°C), and this photograph was taken after 5 min. Scale bar = 2.5 cm. (**C**) A laboratory experiment to quantify this buoyancy disruption by juxtaposing the effect of salinity on copepod buoyancy with their innate swimming response away from light upon increasing intensity in a natural light field. The laboratory apparatus mimicked the underwater angular light distribution (for details see Cohen et al., 2015), modified here to include a 175-W xenon arc lamp (Spectral Products) with a BG-18 broadband blue filter (Melles Griot) that encompasses the wavelength sensitivity of *C. finmarchicus* (Buskey and Swift, 1985; Båtnes *et al*., 2015). Temperature was held at 11.5°C, corresponding to the collection depth in Loch Etive. CV-stage *C. finmarchicus* copepodites were tested in groups of ~ 100 individuals in an acrylic column (5 cm L × 5 cm W × 10 cm H). After a 15-min acclimation period to light and salinity in darkness (D1), light intensity was increased at 5-min intervals (10–16 log photons m^−2^ s) and then returned to darkness for 5 min (D2). The proportion of copepods in the upper half (positive values) and lower half (negative values) of the column were scored from video records at the end of each 5-min interval. The experiment was replicated three times at salinities of 24, 28, 32 and 34 psu. Mean proportions (± SD, *n* = 3 for each salinity level) are plotted, with asterisks denoting significant differences between the ratio of copepods in the upper and lower halves of the column at a given light intensity, relative to the dark control (D1) (one-way RMANOVA, Dunn’s post hoc test, *α* = 0.05) and non-significant data shown (n.s.d.). Pressure was not controlled in these experiments.

Observations of large-scale surface aggregations of *Calanus finmarchicus* and associated marine mammal predators have been well documented for over a century (e.g. Marshall and Orr, 1972). Oceanic fronts (Wishner et al., 1988), and behaviours like swimming in response to light (Folt and Burns, 1999; Cohen and Forward, 2009) are potential proximate factors for aggregations of zooplankton. In addition, the copepod *C. finmarchicus* accumulates large lipid reserves in a conspicuous oil sac (Fig. 1A) that is less dense than seawater with greater compressibility and thermal expansivities (Visser and Jónasdóttir, 1999; Campbell and Dower, 2003; Irigoien, 2004). Many swarming Calanoid copepods possess large oil sacs, which would decrease their effective specific gravity. We propose that the physical properties of the oil sac in relation to specific environmental conditions may lead to *Calanus* surface swarms.

Here, we describe two laboratory experiments, which demonstrate that environmentally relevant salinity and temperature changes can alter buoyancy in *C. finmarchicus* CV copepodids beyond their capacity to regulate vertical position by swimming. Experiments used animals collected from the 50–140-m depth in a sea loch in western Scotland (Loch Etive, 56°45′N, 5°18′W) during September (18th), October (3rd and 23rd) and November (6th and 27th) 2017. The *C. finmarchicus* population is well characterized in this sea loch, with CV copepodids in diapause below 50 m depth in autumn (Clark et al., 2012, 2013; Häfker et al., 2018). At this location, time and depth, CV copepodids show high total lipid content (~129 μg ind^−1^; Clark et al., 2012). The water column was stratified and highly stable throughout the collection period, with an average temperature of 11.41°C (±0.02, SD) and salinity of 27.33 psu (± 0.07, SD) from the 50–140-m depth. The temperature and salinity above 50 m was far more heterogeneous (12.44°C ± 0.88 and 24.08 psu ± 2.86).

## SALINITY CHANGE ALTERS BUOYANCY OF CV-STAGE *C. FINMARCHICUS*

We observed in the laboratory that when freshly collected *C. finmarchicus* CV copepodids, assumed to be acclimatized to the conditions at the 50–140-m depth in Loch Etive, were transferred in the laboratory to less saline water representative of the Loch Etive upper water layer (24 psu), they sank, even when not swimming (Fig. 1B). Conversely, when animals from the same collection were transferred to more saline water representative of shelf and open ocean conditions (34 psu), they floated, even when not swimming. If these copepods possessed oil sac volumes that conferred neutral buoyancy while overwintering (e.g. Pond and Tarling, 2011; Pond, 2012), then observed buoyancy changes from acute decreases and increases in salinity reflect changes in the effective specific gravity of copepods in water of different densities.

**Fig 2:**
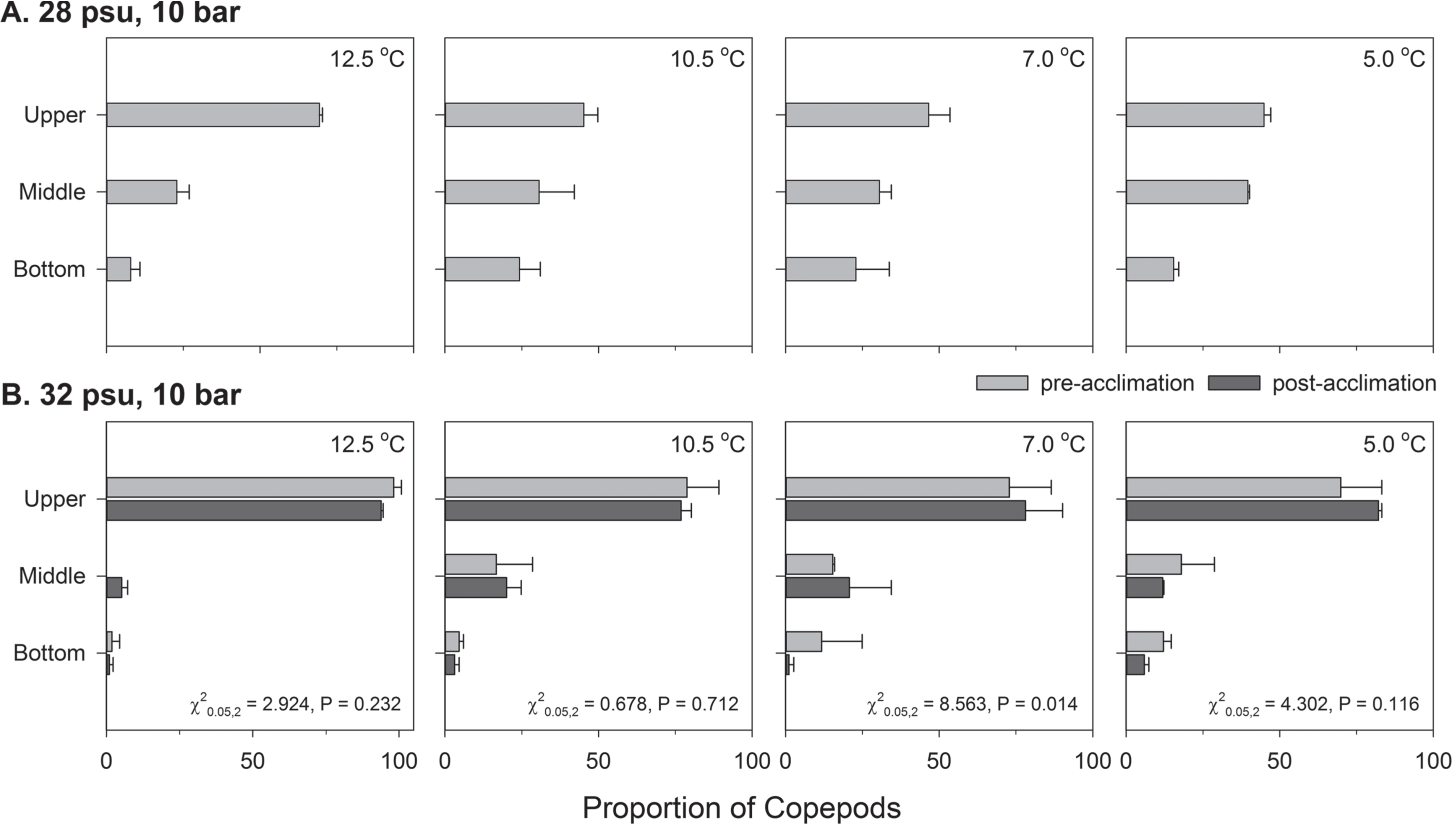
Vertical position of CV-stage *C. finmarchicus* in a pressurized laboratory column across a range of salinity and temperature combinations. This experiment was conducted in darkness. *Pre-acclimation trials* were conducted at 28 psu (A, light gray bars) and 32 psu (B, light gray bars) in GF/F-filtered 50-m-depth Loch Etive water. For the 32-psu treatment, salinity was adjusted with aquarium salt crystals (Tropic Marin sea salt). Copepods (*n* = 49–60) were transferred to treatment water in duplicate glass pressure tubes (250 mL, 21 cm height of potential swimming depth, Ace Glass) within a light-tight water bath with variable temperature control. The Teflon stoppers used to seal the pressure tubes were drilled and equipped with Swagelok fittings to allow pressure control using a nitrogen gas cylinder and low-pressure regulator. Pressure was initially increased from ~ 1 to 10 bar, and temperature held at 12.5°C for 15 min. With pressure maintained at 10 bar, temperature was decreased to 10, 7.0 and 5.0°C, at successive 15-min intervals. The proportion of copepods in the upper third (Upper), middle third (Middle) and bottom third (Bottom) of the columns were scored from video records at the end of each 15-min interval. *Post-acclimation trials* (B, dark gray bars) were conducted with a second copepod collection, repeating the 32-psu trial with animals maintained in treatment water for ~72 h at atmospheric pressure and 11°C prior to increasing pressure to 10 bar and initiating the trial as described above. Means (± SD, *n* = 2) are plotted. A χ^2^ test statistic and associated *P* value comparing 32 psu pre- and post-acclimation at each temperature are provided.

*Calanus* spp. display the innate response to swim away from light in a natural light field (Båtnes et al., 2015) forming the basis of their diel vertical migration behaviour (Cohen and Forward, 2009). Given that *C. finmarchicus* exhibits this strong behavioural response to light, we juxtaposed their negative phototaxis upon increasing light against the effect of salinity on buoyancy (see Fig. 1C for methods). Examining the proportion of copepods in the upper and lower halves of an acrylic rectangular column across different light intensities within four salinity treatments (24, 28, 32 and 34 psu), we confirmed patterns of vertical distribution dependent upon salinity level as described above (Fig. 1C). At salinities of 24 and 28 psu, copepods were more abundant in the lower half of the column in darkness, descending in even greater proportions as light intensity increased (one-way RMANOVA for each salinity; *P* < 0.001 for 24 and 28 psu). In contrast, at 32 and 34 psu the distribution of copepods was skewed toward the upper half of the column. While copepods could descend to some degree against their positive buoyancy in response to light at 32 psu (*P* = 0.008), they remained more abundant in the upper half of the column. However, at 34 psu light had no effect on vertical distribution (*P* = 0.261) with copepods remaining in the upper half of the column demonstrating little ability to swim down.

## HYDROSTATIC PRESSURE, TEMPERATURE AND SALINITY CHANGE ALTER BUOYANCY OF CV-STAGE *C. FINMARCHICUS*

Atmospheric pressure and water temperature both affect water density and may therefore influence *C. finmarchicus* buoyancy at depth. Therefore, we examined the vertical distribution of CV-stage *C. finmarchicus* in laboratory columns under 10 bar pressure with 28- and 32-psu salinity treatments across temperatures from 12.5 to 5.0°C (see Fig. 2 for methods). In preliminary experiments, we found that vertical distributions of copepods in 28 psu were uniform at 10 bar but not at atmospheric pressure (*P* < 0.001, χ^2^ test, data not shown). However, the salinity response we observed at ~ 1 bar (Fig. 1) also occurred at 10 bar, as greater proportions of copepods were present in the upper third of the columns at 32 psu as compared to 28 psu (Fig. 2; *P* < 0.05 for χ^2^ tests of section × salinity, within each temperature level). Acclimation for ~ 72 h to 32 psu did not alter this increase in positive buoyancy (pre- versus post-acclimation, Fig. 2B). Finally, we observed an effect of increased temperature paralleling that of increased salinity, with greater proportions of copepods in the upper third of the columns at 12.5°C as compared to other temperatures (Fig. 2; *P* < 0.05 for χ^2^ tests of section *×* temperature, within each salinity level) presumably due to thermal expansion of lipid reserves (reviewed in Pond, 2012). These differences were not attributable to differences in oil sac size among the three salinity treatments (*P* = 0.658, Kruskal-Wallis ANOVA). Thus, under hydrostatic pressures from depths to which these copepods were acclimatized, rapid increases in salinity and/or temperature result in positive buoyancy and surface entrainment of these animals.

Based on these laboratory results, we propose a working hypothesis that the physical environment—salinity, temperature and hydrodynamics—in conjunction with the biophysical properties of lipids may explain a mechanism for large-scale surface aggregations of copepods in marine environments. Field experiments using Calanoid surface swarms are now required to substantiate this hypothesis.
